# Subsecond lung cancer detection within a heterogeneous background of normal and benign tissue using single-point Raman spectroscopy

**DOI:** 10.1117/1.JBO.28.9.090501

**Published:** 2023-09-09

**Authors:** Frédéric Leblond, Frédérick Dallaire, Trang Tran, Rajeev Yadav, Kelly Aubertin, Eric Goudie, Philippe Romeo, Christopher Kent, Charles Leduc, Moishe Liberman

**Affiliations:** a Polytechnique Montréal, Department of Engineering Physics, Montreal, Québec, Canada; b Centre de Recherche du Centre Hospitalier de l’Université de Montréal, Montréal, Quebec, Canada; c Institut du cancer de Montréal, Montreal, Quebec, Canada; d Reveal Surgical, Montreal, Quebec, Canada; e INSERM UMR_S1109 and Université de Strasbourg, Institut d’immunologie et d’hématologie, Team Tumor Biomechanics, Strasbourg, France; f Centre Hospitalier de l’Université de Montréal, Division of Thoracic Surgery, Montreal, Quebec, Canada; g Centre hospitalier de l’Université de Montréal, Department of Pathology, Montreal, Quebec, Canada

**Keywords:** Raman spectroscopy, lung cancer, bronchoscopy, biopsy, surgery, machine learning, tissue optics, biochemistry

## Abstract

**Significance:**

Lung cancer is the most frequently diagnosed cancer overall and the deadliest cancer in North America. Early diagnosis through current bronchoscopy techniques is limited by poor diagnostic yield and low specificity, especially for lesions located in peripheral pulmonary locations. Even with the emergence of robotic-assisted platforms, bronchoscopy diagnostic yields remain below 80%.

**Aim:**

The aim of this study was to determine whether *in situ* single-point fingerprint (800 to $1700\;{\mathrm{cm}}^{-1}$) Raman spectroscopy coupled with machine learning could detect lung cancer within an otherwise heterogenous background composed of normal tissue and tissue associated with benign conditions, including emphysema and bronchiolitis.

**Approach:**

A Raman spectroscopy probe was used to measure the spectral fingerprint of normal, benign, and cancer lung tissue in 10 patients. Each interrogated specimen was characterized by histology to determine cancer type, i.e., small cell carcinoma or non-small cell carcinoma (adenocarcinoma and squamous cell carcinoma). Biomolecular information was extracted from the fingerprint spectra to identify biomolecular features that can be used for cancer detection.

**Results:**

Supervised machine learning models were trained using leave-one-patient-out cross-validation, showing lung cancer could be detected with a sensitivity of 94% and a specificity of 80%.

**Conclusions:**

This proof of concept demonstrates fingerprint Raman spectroscopy is a promising tool for the detection of lung cancer during diagnostic procedures and can capture biomolecular changes associated with the presence of cancer among a complex heterogeneous background within less than 1 s.

Lung cancer is the most prevalent form of cancer worldwide and the leading cause of cancer-related deaths.^1^ Despite recent advances in treatment, the 5-year survival rate has remained largely unchanged under 20%.^2^^,^^3^ This is largely due to the fact most patients are diagnosed at advanced disease stages where treatments are often ineffective and prognosis is poor. Despite recent advances in treatment, surgical resection remains the mainstay of curative treatment for non-small cell lung carcinoma (NSCLC) and SCLC. Survival rate depends on the ability for the surgeon to remove completely the cancer, which in advanced cases remains below 15% of all treated patients. This low survival rate can be traced back to the variegated tumor response to therapy, the evolution of tumor under treatment pressure and the lack of diagnostic/prognostic biomarkers.^4^ On the other hand, patients treated at early stages of the disease show 5-year survival rates at >70%, placing emphasis on the value of early diagnosis.^2^^,^^5^

Bronchoscopy is used to monitor and screen a variety of conditions, such as bacterial, fungal, and viral infections, as well as bleeding. The technique was also developed to provide fine-needle biopsies and diagnosis of early-stage cancer when standard imaging tests cannot confirm that a pulmonary nodule is benign. Endoscopic approaches have traditionally been limited to large and/or central lung nodules, often prohibiting access to smaller peripheral pulmonary lesions. In those cases, image-guided trans-thoracic needle biopsies can be used despite higher complications rates. Image-guided trans-thoracic needle biopsies use images, such as computed tomography (CT), fluoroscopy—and sometimes ultrasound or magnetic resonance imaging—to help guide the interventional radiologist’s instruments to the site of the abnormal growth. Since most lung nodules are in the lung periphery, new methods were needed to address the technical challenges related to the endoscopic navigation of small peripheral airways.

Advances in bronchoscopy procedures using robotic technology improved diagnostic procedure accuracy, allowing real-time visualization of the bronchial tree deep into peripheral lungs.^6^ Emerging endoluminal robotic platforms allow visual inspection of the airways based on video-rate white-light imaging using flexible catheters. With patients under general anesthesia and in supine position, the catheter is steered by physicians through the mouth into the endotracheal tube and navigated to its target, e.g., a lung nodule. 3D guidance using robotic platforms is achieved using an imaging software based on a pre-determined path set from a pre-procedural CT scan. Target location can be further adjusted to make precise catheter adjustments based on intra-procedural 3D images, e.g., using an external cone-beam CT system.

Despite impressive technological advances, current lung biopsy methods suffer from limitations, including low diagnostic yields and poor specificity.^5^^,^^7^^–^^9^ These factors, combined with patients’ respiratory motion, CT-to-body divergence, and tumor heterogeneity, lead to an inability for clinicians to properly locate biopsy sites within the neoplastic tissue and negatively affect diagnostic yields. Recent studies demonstrated robotic-assisted bronchoscopy allowed physicians to navigate deep into the peripheral lungs, but still suffered from low diagnostic yields. In a retrospective study using the Monarch robotic-assisted bronchoscopy platform from Johnson & Johnson (167 lesions and 165 patients), tissue biopsy was successfully obtained in 97.6% of patients, with an overall diagnostic yield of 69.1% to 77%.^10^ This study included 13 biopsy sites that were non-diagnostic and were associated with inflammation. In a prospective multi-centered study with the same system (54 patients and 5 medical centers), the diagnostic yield was 74.1%.^11^ In a retrospective single-center study using the ion robotic-assisted bronchoscopy platform from intuitive surgical (130 patients and 159 lesions), successful navigation was achieved in 98.7% of lesions. The study found an overall diagnostic yield of 81.7%, with a 70.9% yield in lesions located in the lung periphery.^12^

Thus, procedures designed to improve early diagnosis of lung cancers could greatly benefit from an adjunct tool capable of providing non-destructive, spatially precise, and real-time identification of cancerous tissue with high sensitivity and specificity. Raman spectroscopy is a molecular imaging technique that can reliably detect a range of biological indicators of cancer without the need for any labeling or contrast agents. Relying on the intrinsic inelastic light scattering properties of tissue, it is well suited to intraoperative *in situ* use during diagnostic procedures as it is non-destructive and leaves tissue unmodified after analysis.^13^^,^^14^ By detecting inelastically scattered laser light, the technique provides a molecular signature based on the vibrational modes associated with chemical bonds in tissue, which can allow for quantitative and specific information of tissue characteristics in biomolecules, including lipids, DNA, proteins, and specific aromatic amino acids.^15^ The technique can be implemented for remote detection using flexible optical fibers that are compatible with tissue excitation and detection of scattered light through the inner cannula of commercial bronchoscopes.

The Zeng group pioneered the development and clinical use of fiber optics single-point Raman spectroscopy probes for the detection of lung cancer. Their earliest work in 2003 demonstrated—in specimens form 10 patients—that the Raman spectral fingerprint (700 to $1800\;{\mathrm{cm}}^{-1}$) could be used to distinguish normal lung tissue from NSCLC—adenocarcinoma, squamous cell carcinoma—using spectroscopic measurements that took 5 s to acquire.^16^ In later work, they demonstrated clinical systems based on a flexible endoscopic Raman system that can acquire high wavenumber spectra. In 2011, pre-neoplastic lesions were detected with 96% sensitivity and 91% specificity—in 26 patients—using spectra ranging from 1550 to $3100\;{\mathrm{cm}}^{-1}$. In a more recent 2017 study, they demonstrated *in situ* detection of high-grade dysplasia and *in situ* carcinoma—in 80 patients—with 90% sensitivity and 65% specificity. The intra-procedural Raman spectroscopy catheter that was used by the Zeng group relied on high wavenumber spectra (2775 to $3400\;{\mathrm{cm}}^{-1}$) and led to measurements acquired in 1 s. This technological choice (high wavenumber rather than fingerprint region of a Raman spectrum) was made because they reported intraprocedural fingerprint signals—despite being associated with richer bio-informational content—had limited Raman-to-fluorescence signal-to-background ratios leading to prohibitively long imaging times.^17^

Here, a study is presented that was designed to evaluate fingerprint *in situ* Raman spectroscopy for its potential to differentiate malignant lung tissues from tissue that is either normal or associated with benign inflammatory conditions, including emphysema and pulmonary bronchiolitis. The dataset presented here was collected in 2018 with an early version of the intraoperative system developed by the company Reveal Surgical.^18^ The instrument is portable and relies on a hand-held probe that was recently repackaged (for intra-procedural use) into a flexible endoscope, with <2  mm outer diameter compatible with commercial bronchoscopy systems. When used in direct contact with tissue, the probe provides a spectrum from a circular area 0.5 mm in diameter, with depth sampling ranging up to <500 micrometers, depending on tissue albedo.^19^ The probe has an outer diameter of 2.1 mm and is connected to a spectrometer through a cable composed of low hydroxyl content multi-mode fibers: eight fibers with 300  μm core are used for detection and a central 300  μm core fiber is dedicated to tissue excitation at 785 nm. At the tip of the probe, a lens is used to ensure co-location of excitation and detection spots, and a ring-shaped short-pass filter is used to remove elastic scattering at the laser excitation wavelength and to minimize the Raman and fluorescence signal from silica fibers.^20^

Recruited patients were men (80%) and women (20%) with primary lung cancer undergoing thoracotomy. Each patient was monitored by the CHUM Ethics Committee. The cohort included 10 patients with different cancer types or grades and for whom surgery was the first line of treatment, with absence of neoadjuvant therapy. Demographic and clinico-pathological details relating to the patient cohort are reported, including smoking habits, age, sex, tumor localization, cancer type, and pathological stage (Table 1). The Raman spectroscopy system was used to characterize fresh human lung tissue specimens from all patients. The handheld probe was put in direct contact with excised tissue to acquire spectra. Before each measurement, the system charge-coupled device (CCD) sensor was cooled to −80°C and the probe connected to the portable spectroscopy system. Calibration of the Raman shift axis (x-axis of each spectrum) was determined from a spectrum acquired using acetaminophen powder (Tylenol^®^) prior to each measurement. The system response was characterized using the fluorescence spectrum of a standard reference material (SRM 2214, NIST, United States). For each measurement, the probe was placed in contact with the specimen and a dark count measurement taken with the laser off (integration time of 50 ms). Then, a series of 10 repeat Raman measurements were acquired with an integration time of 75 ms. The laser power was manually adjusted for each measurement up to 150 mW (at the tip of the probe) to optimize the overall photon counts while ensuring no camera saturation resulted. After each measurement, a collocated biopsy sample was harvested for histopathology analysis. For each patient, the surgeon identified areas of normal/benign and cancer/necrotic tissue; up to 10 measurements were done in normal/benign and cancer/necrotic tissue, for a maximum of 20 measurements per patient. A biopsy sample was harvested co-located with the spectroscopy measurement. The samples were formalin-fixed and paraffin-embedded and hematoxylin & eosin (H&E) stained for diagnostic confirmation by a pathologist.

**Table 1:** Demographic, clinical, and pathological characteristics of all patients. Number of samples retained after rejecting lower quality spectra are shown in parentheses.

Number of patients	10
Median age (standard deviation)	70 (8)
Male/female	8/2
Normal/benign samples	—
Lung with no abnormalities	24 (17)
Emphysema/pulmonary bronchiolitis	76 (53)
Cancer samples	—
Non-small cell carcinoma	—
Adenocarcinoma	32 (16)
Squamous cell carcinoma	17 (5)
Not otherwise specified	20 (15)
Small cell carcinoma	10 (10)
Necrosis samples	18 (16)

A total of 197 spectral fingerprints (100 in normal/benign tissue and 97 in cancer/necrosis) were acquired. Each spectrum was correlated with the correspondent histopathological characteristics: normal lung or benign tissue (emphysema and bronchiolitis); cancer (adenocarcinoma, squamous cell carcinoma, other NSCLC, SCLC) or necrosis (Table 1). Several processing steps were applied to each raw spectrum to isolate the vibrational spectroscopy contribution using a custom software.^21^ Those included: averaging of the 10 repeat spectra, subtraction of dark count spectrum acquired with the laser turned off, normalization with the NIST standard to correct for the instrument response, x-axis (wavenumber shift) calibration, removal of background signals using the custom background removal algorithm BubbleFill, and standard normal variate (SNV) normalization.

The quality factor (QF) metric was then computed from each resulting normalized spectrum.^21^ The QF provides an assessment of the likelihood each SNV-normalized spectrum is associated with tissue Raman peaks or stochastic noise [Fig. 1(a)]: values close to 1 were unlikely to be of random nature while values closer to 0 would be mostly noise. Average SNV-normalized spectra—for cancer (including necrosis) and non-cancer (normal/benign)—are shown, with the large inter-measurement variance hinting at the presence of highly noisy spectral data points contaminating the dataset [Fig. 1(d)]. The same analysis was done, this time using only spectra with a QF metric larger than 0.4 [Fig. 1(f)]. This resulted in the rejection of 33% of all measurements made. All rejected spectra were visually inspected: the vast majority were noisy, in most cases not clearly showing the ubiquitous Raman peaks encountered in biological tissue (e.g., amide bands and phenylalanine peak). Rejecting spectra based on the QF metric only minimally affected the balance between classes: the ratio of the numbers of cancer/necrosis over normal/benign samples was 0.97 before the cutoff and 0.89 after. The spectral fingerprint for each individual tissue type (adenocarcinoma, squamous cell carcinoma, not otherwise specified NSCLC, SCLC, necrosis, and normal/benign) is shown for QF>0.4 along with representative histology images (Fig. 2). None of the spectra associated with the first patient of the study had a QF larger than 0.4.

**Fig. 1:**
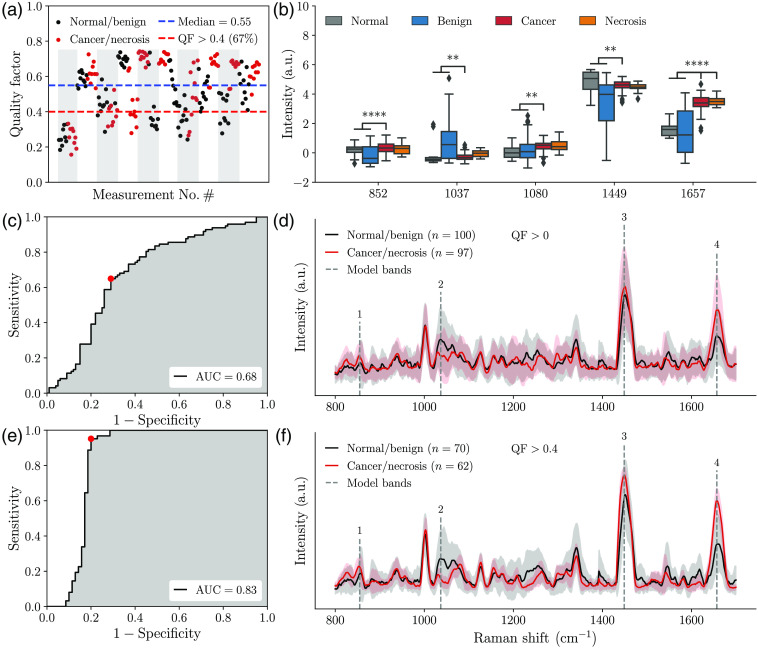
(a) QF metric computed for all spectral fingerprint measurements acquired in normal/benign and cancer/necrotic lung tissue. The x-axis represents all measurements acquired in chronological order for all 10 patients, which are denoted by the alternating gray and white bands. (b) Whisker plots associated with the four bands selected for training the normal/benign versus cancer/necrosis machine learning models and the hemoglobin band at $1080\;{\mathrm{cm}}^{-1}$. Statistical significance is represented by ** for p<0.01 and **** for p<0.0001. (c) ROC curve for the cancer detection model trained using the whole dataset and (d) corresponding average Raman spectra with inter-measurements variance shown. (e) ROC curve for the cancer detection model trained using only high-quality spectral (QF > 0.4) and (f) corresponding average Raman spectra with inter-measurements variance shown. The four bands selected for model training are shown with gray dotted lines in panels (d) and (f).

**Fig. 2:**
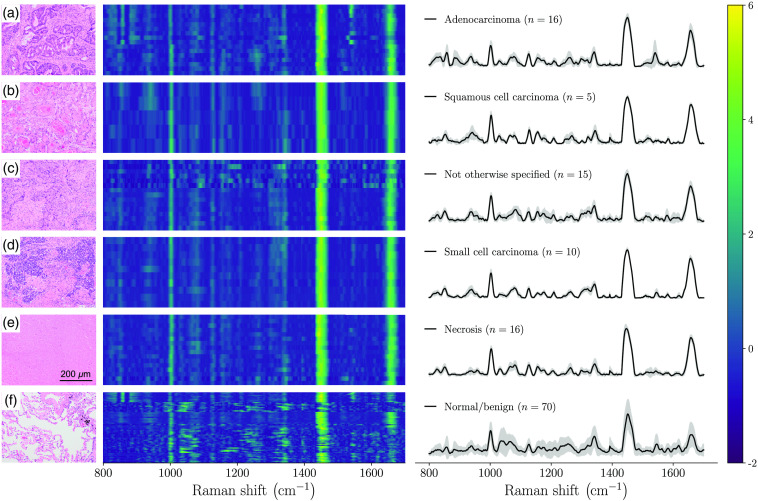
Representative histology images (left, H&E-stained tissue slices), Raman spectrograms (middle), and average SNV-normalized Raman spectra with inter-measurements variance (right): (a) adenocarcinoma, (b) squamous cell carcinoma, (c) non-small cell cancer of unspecified origin, (d) small cell carcinoma, (e) necrosis, and (f) benign lung tissue. Only the spectra with QF>0.4 are shown. Spectrograms show all acquired spectra with QF>0.4 on the same image.

Two machine learning models were then developed for the detection of cancer/necrosis from normal/benign tissue, either using the whole dataset [97 cancer/necrosis samples and 100 normal/benign samples (Fig. S1 in the Supplementary Material] or only data with QF>0.4 [62 cancer/necrosis samples and 70 normal/benign samples (Fig. 2)]. Prior to machine learning model training and validation, the number of features (i.e., all intensity value within a spectrum) was reduced to include only those that contributed the most to the variance between cancer/necrosis and non-cancer tissue. This was accomplished using linear support vector machines (SVM) with L1 regularization.^22^ Machine learning model training from the dimensionally reduced features set was then done using linear SVM with the regularization parameter C. Each time a model was trained, hyperparameters (number of features, C) were selected by carrying out a grid search across all possible combinations. The regularization parameter C was varied between 0.01 and 5, the number of individual bands was varied between 5 and 20. For each combination, performance was assessed using leave-one-patient-out (i.e., 10-fold) cross validation based on the number of false/true positives and false/true negatives, by comparing the model prediction with the assigned pathological label. Accuracy, sensitivity, and specificity were calculated from a receiver-operating-characteristic (ROC) curve analysis, and the area under curve (AUC) was reported. In this approach, all spectra from one patient were used as a validation set, while all remaining spectra were used to train a model. The features that were retained for machine learning model building are represented by numbered dotted lines in Figs. 1(d) and 1(f). The spectral region from 1060 to $1100\;{\mathrm{cm}}^{-1}$ was excluded from the feature selection process since it may have been associated with blood that had contaminated the surface of the specimens.^23^^,^^24^

The model trained from the whole dataset led to a ROC curve with an AUC of 0.68, corresponding to a sensitivity of 71% and a specificity of 65%. However, the model trained using only high-quality data (QF>0.4) resulted in an AUC of 0.83, corresponding to a sensitivity of 94% and a specificity of 80%. To test whether those results were associated with chance, N=100 models were trained with the same spectra but with the histology labels randomly assigned. Using the same hyperparameters as above, this resulted in an average AUC of 0.47±0.03. This provides evidence that the reported performance associated with the QF>0.4 model did not result from over-fitting of the data.

The main Raman bands that were detected in this study were consistent with those from the 2003 Huang study up to minor wavenumber (x-axis) shifts (Table 2).^16^ That study distinguished tumor (adenocarcinoma and squamous cell carcinoma) from normal bronchial tissue but did not present machine learning models and did not include emphysema and bronchiolitis specimens. The most statistically significant biomolecular differences—associated with those bands between normal/benign and cancer/necrosis—were identified based on univariate statistical analysis. Overall, five peaks showed significant differences (p<0.01) and are shown as whisker plots evidencing changes between tissue classes [Fig. 1(b)]. However, all features selected through machine learning modeling were associated with features associated with the bands at 1657 and $852\;{\mathrm{cm}}^{-1}$. Those can be assigned to the amide I band of proteins and possibly a peak associated with the amino acid tyrosine, respectively.^15^ The amide I band had a higher intensity in cancer/necrotic tissue when compared to the categories associated with normal and benign tissue [Fig. 2(b)]. A similar albeit more subtle effect is observed for tyrosine at $852\;{\mathrm{cm}}^{-1}$.

**Table 2:** A comparison of major Raman bands observed in normal/benign and cancer/necrosis bronchial tissues from our study and from Huang et al.,^16^ and a tentative assignment.

Peak No. (Fig. 1)	Band position (${\mathrm{cm}}^{-1}$)	Band position (${\mathrm{cm}}^{-1}$) (Huang et al.^16^)	Main vibrational modes	Tentative biomolecular assignment
1	852	855	Tyrosine ring breathing	Protein (tyrosine)
2	1037	1031	C–H bending	Phenylalanine
	1080	1078	Phosphate vibrations	Blood plasma
	1156	1152	Carotenoid peaks	Protein and blood plasma
3	1449	1445	δCH2 and δCH3	Phospholipids and saturated fatty acids
	1549	1552	Single tryptophan residue	Blood plasma (hemoglobin)
4	1657	1655	Protein (amide I) and lipid [unsaturated (C=C)]	Proteins

The aim of this study was to determine whether Raman spectroscopy using a single-point handheld device could show sufficient sensitivity and specificity for identification of cancerous lesions in freshly excised bronchial tissue. The acquisition time per interrogation point was less than 1 s and the classification resulted in distinguishing normal/benign from cancer tissue with both high sensitivity and specificity. These preliminary results, albeit in a cohort of patient with limited size, provide enticing evidence that the technique could be utilized for real-time spectral acquisition and tissue classification for lung cancer diagnostics in the bronchoscopy surgical workflow. Moreover, to our knowledge, this is the first study reporting lung cancer detection models against a highly heterogenous background not only composed of normal lung tissue but also of tissue associated with benign conditions, including emphysema and bronchiolitis.

Several aspects of this study could be improved to limit the number of false negatives and improve cancer detection specificity. For example, while the model performed well at detecting cancer, the spectra associated with normal/benign tissue remained noisy within those peaks selected for tissue classification, namely around $852\;{\mathrm{cm}}^{-1}$ and at $1657\;{\mathrm{cm}}^{-1}$. While limiting the number of features to two bands ensured the model did not overfit the data, it made the model more sensitive to noise. This study could not reveal what fraction of this “noise” was biological (i.e., intrinsically associated with the heterogeneity of the normal/benign background) and what fraction was associated with stochastic photonic noise. Future studies should be conducted optimizing acquisition parameters (laser power, integration time, and number of accumulations) to further increase the QF factor thereby ensuring the impact of photonic noise is minimized. This should be done also to ensure all spectra have sufficient spectral quality and all spectra data collected *in situ* intra-procedurally meet a minimum QF value.

The true value of Raman spectroscopy applied to lung tissue characterization will come from machine learning models developed from larger scale datasets (e.g., at least 50 to 100 patients and more than 1000 spectral data points) with high spectral quality. This will allow the use of many features from the fingerprint Raman spectra toward the development of generalizable models tested on independent hold-out datasets. Careful study designs will be required to ensure models can be trained from data fully capturing the heterogeneity of the normal/benign backgrounds to minimize the number of false positives. This approach could revolutionize robotic-assisted bronchoscopy, effectively providing the means to improve diagnostic yields by eliminating non-diagnostic specimens (e.g., misconstruing atelectasis for solid pulmonary nodules and interpreting inflammation as cancer) and diminishing the vulnerability to CT-to-body divergence.

## Code, Data, and Materials Availability

The data and materials information that support the findings of this study are available from the corresponding author upon reasonable request.
