# Supplementary material for: Subsecond lung cancer detection within a heterogeneous background of normal and benign tissue using single-point Raman spectroscopy
Source: J Biomed Opt. 2023 Sep 9;28(9):090501. doi: 10.1117/1.JBO.28.9.090501 (PMC10491897; doi:10.1117/1.JBO.28.9.090501)
Supplement: Supplementary file 1 [file JBO_028_090501_SD001.pdf]

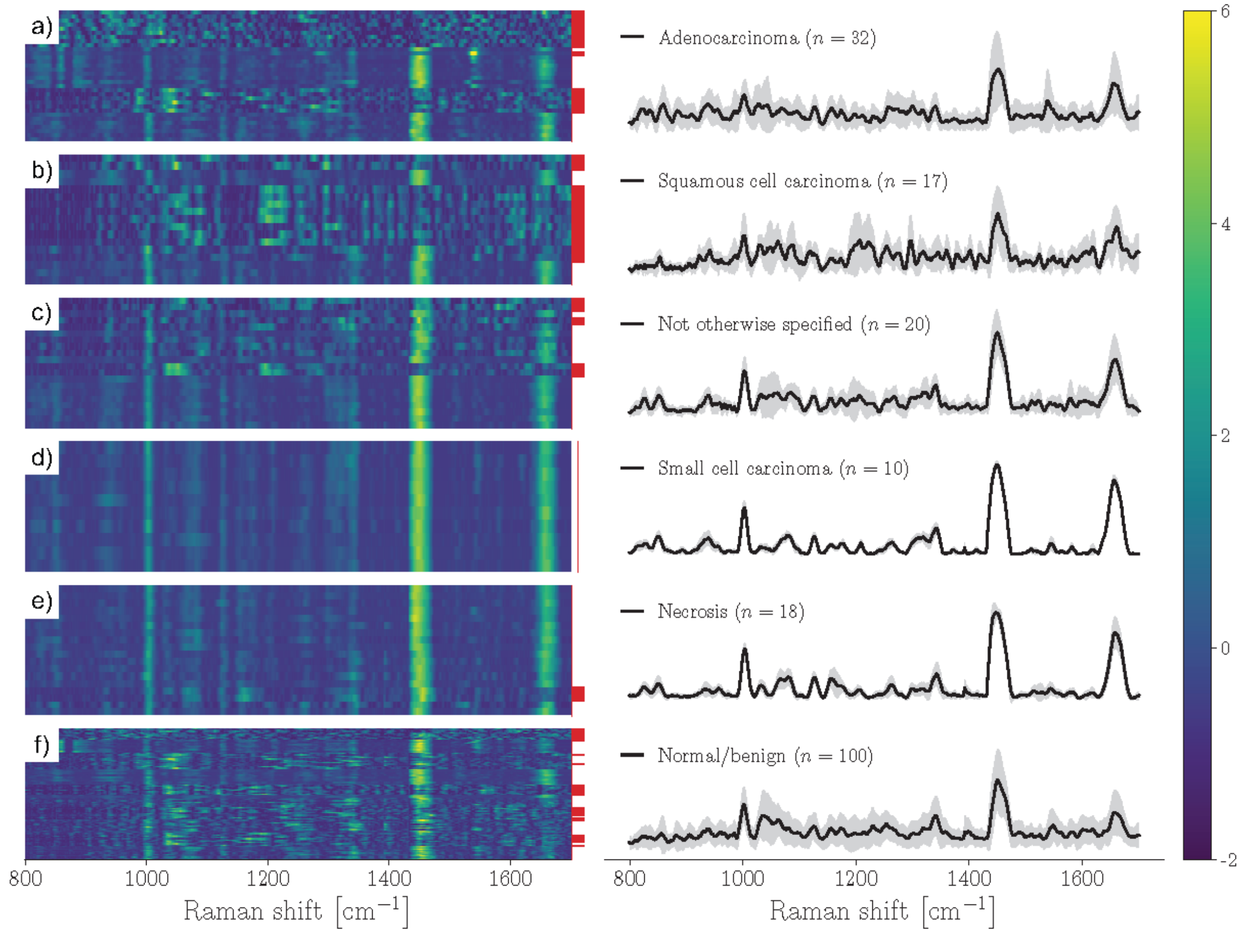

Fig. S1 Raman spectrograms (left) and average Raman spectra with inter-measurements variance (right) for all measurements acquired (no QF cut-off): (a) adenocarcinoma, (b) squamous cell carcinoma, (c) non-small cell cancer of unspecified origin, (d) small cell carcinoma, (e) necrosis, and (f) benign lung tissue. Spectra that were removed to train the models with QF > 0.4 are denoted with a red mark.
